# Carotid web: a little-known etiology of recurrent stroke - case report

**DOI:** 10.1590/1677-5449.202401262

**Published:** 2025-03-14

**Authors:** Nathalia Santana Moreda, Henrique Alves de Almeida, Vinicius Tadeu Ramos da Silva Grillo

**Affiliations:** 1 Centro Universitário São Lucas – UNISL, Porto Velho, RO, Brasil.; 2 Instituto Vascular e Endovascular de Rondônia – IVER, Porto Velho, RO, Brasil.

**Keywords:** carotid artery diseases, ischemic stroke, carotid endarterectomy

## Abstract

Carotid web is a rare and poorly understood condition associated with cryptogenic and recurrent acute ischemic stroke in young patients without atherosclerotic risk factors. We describe the case of a 58-year-old female patient with hypertension and dyslipidemia who had recurrent ischemic strokes for 5 years. Presence of carotid web was suggested by Doppler ultrasonography and confirmed by digital subtraction angiography. The patient underwent endarterectomy and, due to satisfactory internal carotid reflux, it was decided not to use a temporary vascular shunt. Endarterectomy and arteriorrhaphy with a bovine pericardium patch were performed. Although carotid web can be seen with imaging exams, detection can be challenging and it can mimic other conditions, such as arterial dissection, non-calcified atherosclerotic plaque, and intraluminal thrombus. The ease with which it can go unnoticed or misdiagnosed highlights the need to understand this disease.

## INTRODUCTION

It is crucial to determine the etiology of acute ischemic stroke in order to guide strategies for treatment and secondary prevention. Cryptogenic ischemic stroke is a significant challenge for specialists, accounting for approximately 25% of all ischemic stroke cases. This subtype of cerebral vascular accident has an elevated cumulative risk of recurrence, at 21.3%, which indicates that the underlying cause remains.^1,2^

A carotid web is a rarely and poorly understood condition that is associated with cryptogenic and recurrent ischemic stroke in young patients with no atherosclerotic risk factors.^1,3,4^ This pathology constitutes an uncommon form of focal fibromuscular dysplasia, characterized by the abnormal projection of a membranous septum into the internal layer of the carotid, located on the posterior surface of the carotid bulb and the internal carotid artery.^3-5^

The septum formed by the carotid web causes hemodynamic disorders that induce flow stagnation, creating the conditions for formation of thrombi and culminating in cerebral emboli. These conditions show that presence of a carotid web can stimulate thrombogenesis, increasing the risk of ischemic stroke.^1,3,6^

The prevalence of carotid webs in the general population is unknown, but among patients with ischemic stroke it varies from 1.2% to 2.7%. Incidence is 8.9% among transient ischemic attack (TIA) patients, 1.1% among patients with strokes due to occlusion of major vessels, and 4.4% in patients who undergo endarterectomy due to carotid stenosis.^2,3,7,8^

Carotid web is more common among females, accounting for 61.7 to 71% of cases. It predominantly affects the black population, with up to 75% of cases, especially young women with a mean age of 50 years.^3,9-11^ Data from a recent systematic review revealed that carotid web occurs bilaterally in 23.0% of cases, with a majority located on the posterior wall (87.3%) of the carotid artery (98.7%). Additionally, 31.6% of the patients had thrombus adhering to the web, and 75.9% had occlusion of major vessels.^12^

## CASE REPORT 

We present the case of a 58-year-old female patient with a history of systemic arterial hypertension and dyslipidemia going back a decade and who had undergone bariatric surgery. From 2017 to 2022, she had recurrent episodes of ischemic stroke in the territory of the right internal carotid artery (RICA).

During the ischemic episodes, the patient underwent extensive supplementary investigations, including intracranial magnetic resonance and computed tomography, Doppler ultrasonography (USD) of the carotid arteries, and echocardiography, looking for emboligenic foci. However, no significant abnormalities were found and the events were considered to have cryptogenic etiology.

**Figure 1:**
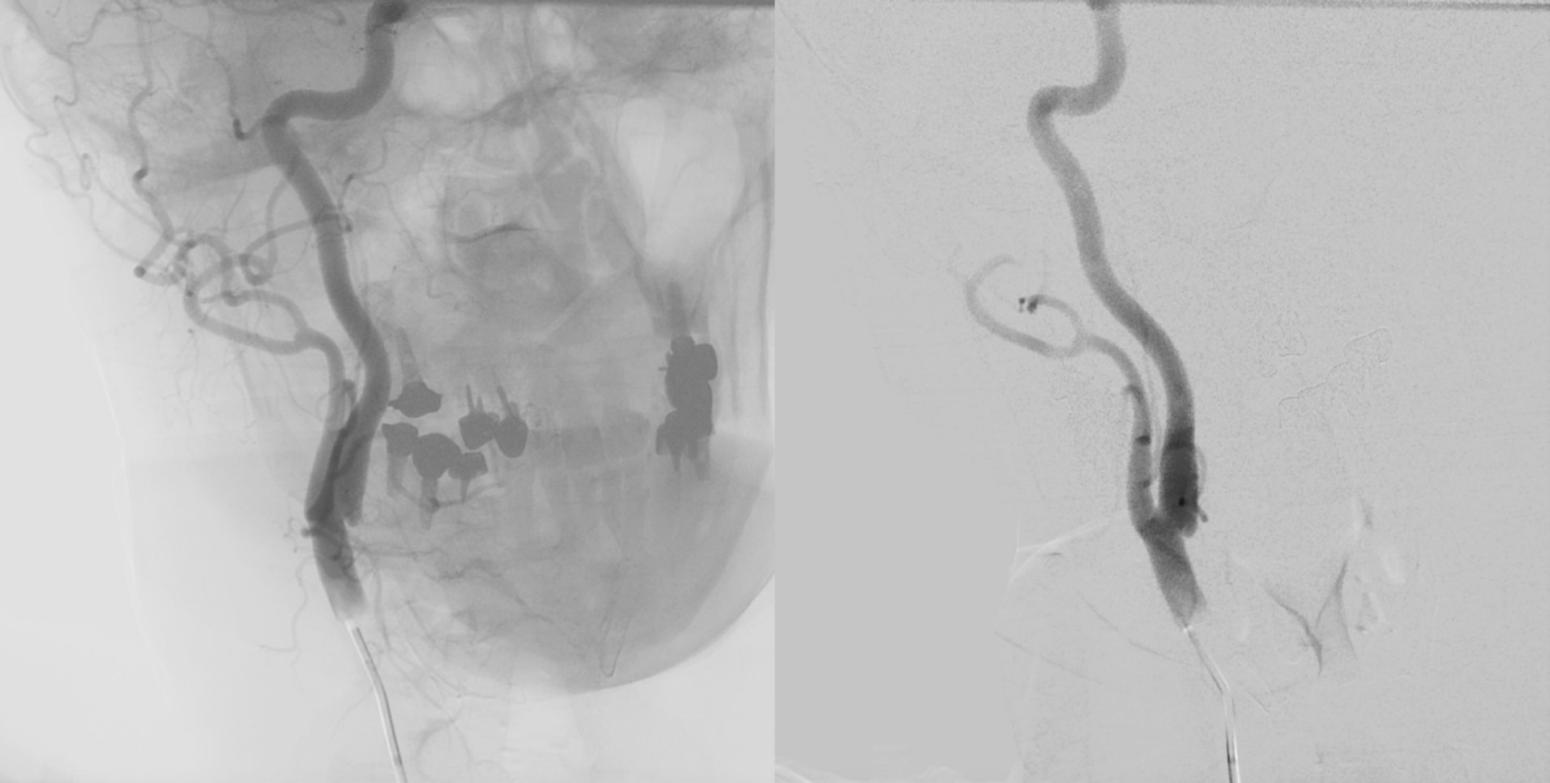
Digital subtraction angiography with the catheter positioned in the right common carotid artery, showing the carotid web, which starts in the common carotid and extends to the origin of the internal carotid. On the right: angiography in anteroposterior view with bone landmarks. On the left: angiography with no bone landmarks.

In 2023, additional supplementary investigations were conducted, including another USD scan of the carotid arteries, which showed images suggestive of a carotid web on the posterior wall of the proximal segment of the RICA. To confirm this diagnosis, the patient underwent digital subtraction angiography (DSA) with a three-dimensional rotational sequence, which confirmed presence of a carotid web in the RICA (Figure 1). This was located on the posterior wall and projected into the vessel lumen, causing filling failure at the site and reducing vascularization of the ipsilateral middle cerebral artery territory.

**Figure 2:**
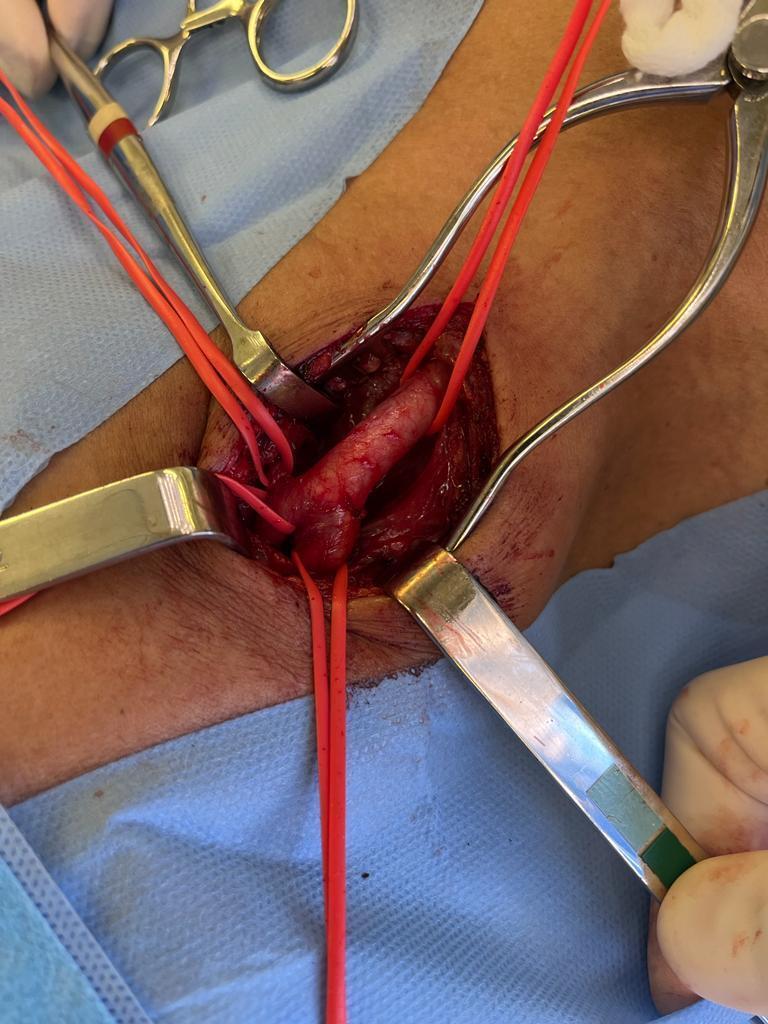
Intraoperative image after dissection and repair with vessel loops around the common carotid artery (on the right of the image), the external carotid artery (top left of the image), superior thyroid artery (top of the image), and internal carotid artery (lower part of the image).

After a surgical risk assessment that classified the patient as low risk, she underwent endarterectomy of the RICA. The procedure was conducted under general anesthesia with oral endotracheal intubation and antibiotic prophylaxis with first-generation cephalosporin. The procedure began with a right-side longitudinal cervicotomy followed by dissection and repair of the common carotid artery (CCA), internal carotid artery (ICA), and external carotid artery (Figure 2). Systemic heparinization was given with 5,000 UI of intravenous unfractionated heparin.

**Figure 3:**
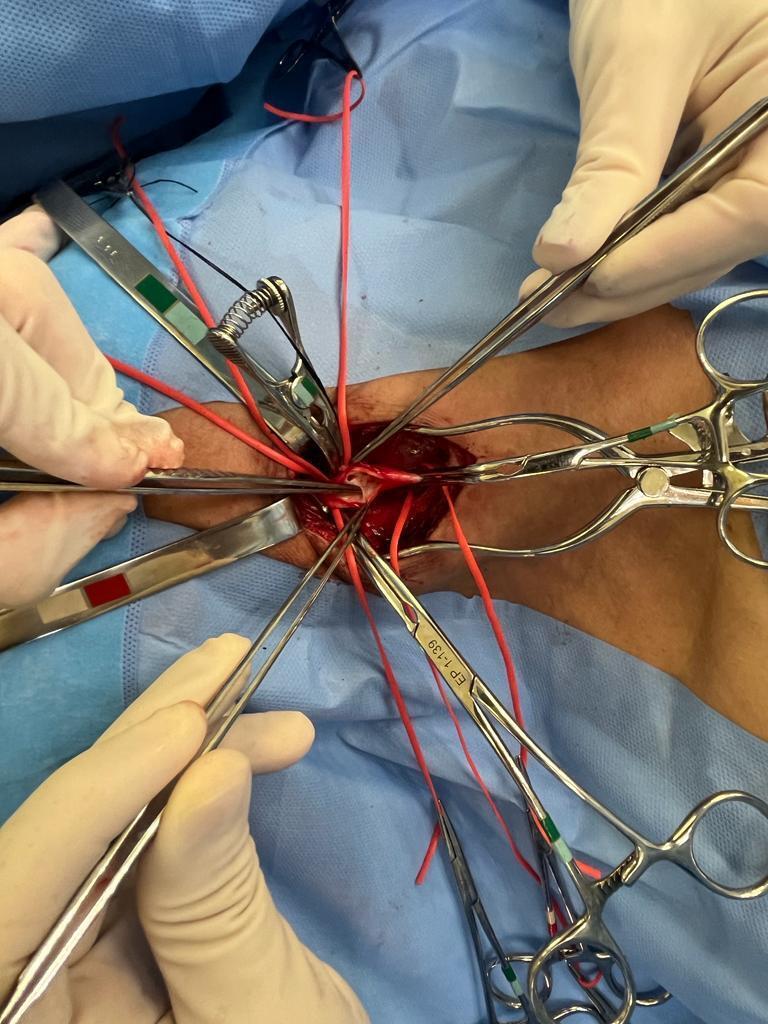
Intraoperative image after clamping the internal and external common carotid artery and arteriorrhaphy. Observe the membranous flap in the carotid bulb.

**Figure 4:**
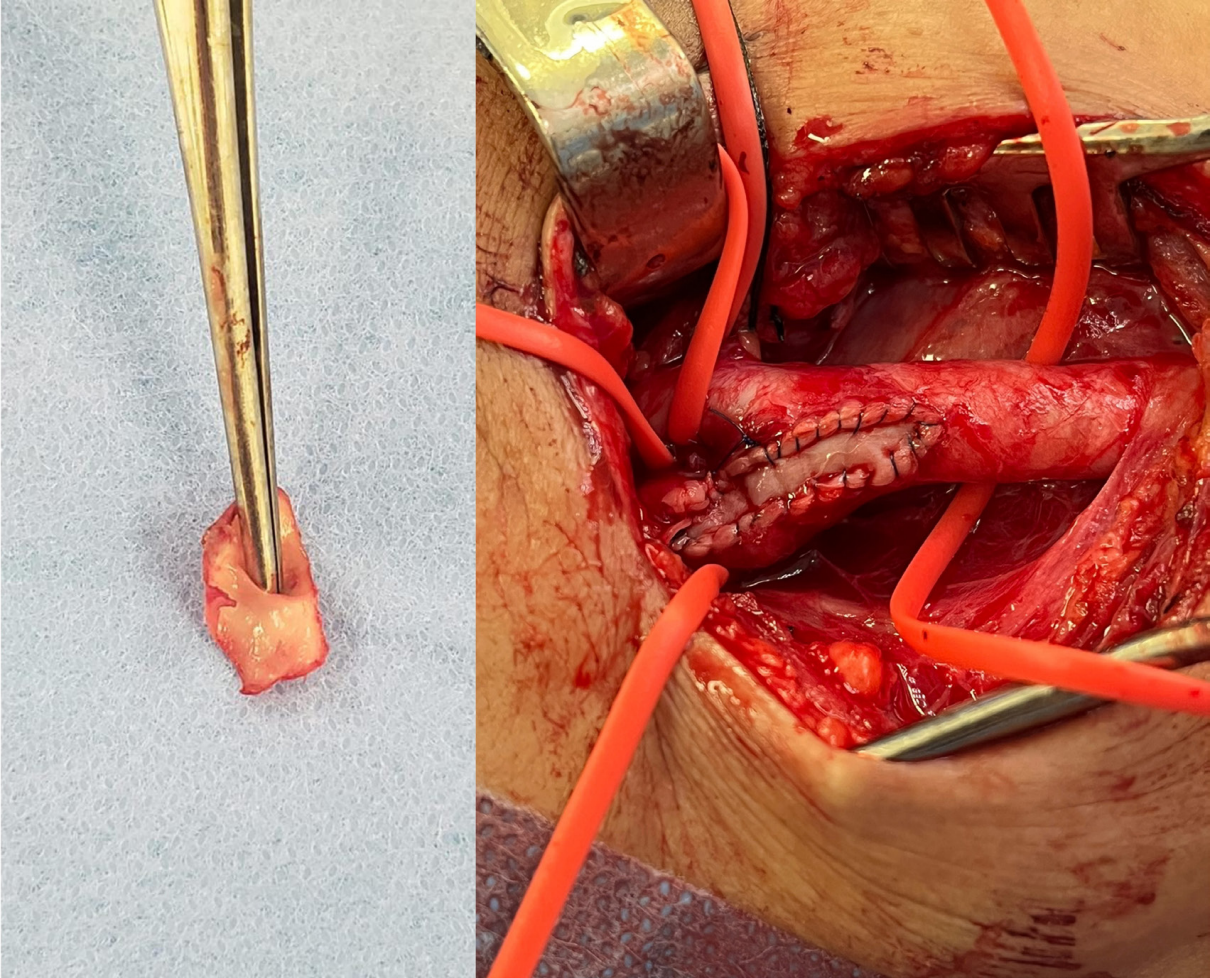
Intraoperative images. On the left: carotid web resected. On the right: arteriorrhaphy with a bovine pericardium patch.

Arteriotomy was performed from the CCA to the ICA and since there was satisfactory reflux from the ICA the decision was taken not to employ a temporary vascular shunt. During the arteriotomy, it was possible to identify the membranous flap within the carotid bulb (Figure 3). Endarterectomy was performed and the carotid web was removed before using a bovine pericardium patch for arteriorrhaphy(Figure 4).

The patient was transferred to the intensive care unit (ICU) for postoperative care, free from neurological changes or complications involving the surgical site. After 1 day in the ICU, she was transferred to a bed in the ward where she remained for 1 more day before discharge from hospital in an excellent general state. At follow-up assessments she remained asymptomatic from a neurological point of view and control USD showed patent carotid arteries.

**Figure 5:**
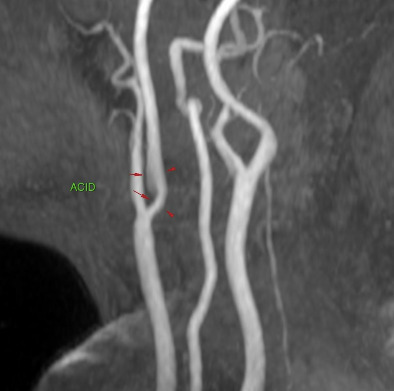
Arterial magnetic resonance angiography of the cervical vessels 1 year after surgery. Note that the right internal carotid artery remains patent, with a discrete reduction in caliber, but without causing significant stenosis.

Arterial magnetic resonance angiography of the cervical vessels conducted 1 year after surgery showed an area of frontotemporal encephalomalacia on the right compatible with sequelae from the previous ischemic events. The RICA remained patent, with discrete reduction in caliber that did not constitute significant stenosis, compatible with her postoperative status (Figure 5).

## DISCUSSION

Initially this patient’s stroke etiology was classified as cryptogenic. The incidence of cryptogenic stroke associated with carotid web was 3.8/100,000 people-years (3.2 in men and 4.3 in women) and the incidence of carotid web was 24 times greater among patients with ischemic stroke involving the anterior circulation of a single hemisphere than among control patients.^1^ Another study observed a statistically significant association between carotid web and ischemic stroke, with a prevalence of 21.2% in the population with cryptogenic stroke, compared to 1.6% in the control group, producing a likelihood ratio of 10.6.^11^

Moreover, in the context of this case report, the occurrence of recurrent ischemic strokes was notable, since the patient was young and had few risk factors for atheromatosis. Carotid web appeared to be an important suspicion factor, since it is associated with a five times greater risk of stroke recurrence compared with cases without this condition.^5^ Moreover, the habitual risk factors seen in the majority of stroke cases were less prevalent among cases of stroke secondary to carotid web, with hypertension in 28.6%, hyperlipidemia in 14.6%, diabetes mellitus in 7.0%, and smoking in 19.8%.^9^

Nonetheless, diagnostic confusion may occur, even when investigations are conducted by experienced professionals, because of the rarity of occurrence of carotid web, which is frequently confused with spontaneous arterial dissection and atherosclerotic plaques.^3^ This diagnostic complexity highlights the need for a careful approach and for consideration of less common causes in cases with atypical presentations, underscoring the importance of detailed knowledge of the distinctive characteristics of carotid web to achieve a more precise diagnostic assessment.

USD of the carotids enables the lumens to be visualized and the carotid web to be identified in cases with or without significant stenosis. However, computed tomography angiography (CTA) and DSA are more advantageous tools for detection of carotid web, since they enable observation of stagnation of the contrast in the membranous septum, even in the late venous phase.^1,3,12^

Although a carotid web can be seen using CTA and DSA, detection can still be challenging, since it generally does not cause hemodynamically significant stenosis. Additionally, carotid web can mimic other conditions, such as arterial dissection, non-calcified atherosclerotic plaque, and intraluminal thrombus.^1,12^ In the case in question, the patient underwent intracranial imaging exams, which did not include assessment of the cervical vessels, possibly contributing to the delayed diagnosis. Sajedi et al.^11^ emphasize the advantages of CTA, because of the noninvasive and rapid acquisition of high resolution images, in addition to its additional benefits for characterization of superimposed findings and its utility for ruling out atherosclerosis or vascular injuries; so they recommend that routine assessment of patients with cryptogenic stroke should include CTA imaging of the neck.

The best treatment approach for carotid web has not yet been defined on the basis of robust evidence, although it is recognized that drug-based treatment may not be enough to reduce the recurrent ischemic events.^3-5^

The patient in this clinical case was successfully treated with carotid endarterectomy, demonstrating that it is a feasible option for treatment of carotid web. A recent bibliographic review compared medical treatment with interventional treatment in 289 patients with carotid web, finding that 52.2% underwent intervention (angioplasty or endarterectomy), while 47.8% were treated clinically (antiaggregation or anticoagulation). There was no perioperative mortality in the intervention group and major complications were reported in 0.5%, with no recurrent ischemic events during follow-up of 3 to 60 months. In contrast, in the medical treatment group, the rate of recurrence of cerebral ischemia over 2 to 55 months was significantly higher, at 26.8%.^9^

## CONCLUSIONS

Carotid web is a rare anomaly that is challenging to diagnose and stands out as a condition that increases the risk of ischemic stroke, primarily recurrent strokes and among young patients without atherosclerotic risk factors.

Rapid diagnostic elucidation is of fundamental importance for optimal prognosis, considering the potential severity associated with the pathology. The ease with which it can pass unnoticed or be diagnosed late underscores the imperative need for health professionals to study this disease and improve their knowledge and understanding of it. Individualized investigation and mastery of supplementary examinations play a crucial role in early diagnosis and effective treatment of this complex condition.
