# Supplementary material for: Carotid web: a little-known etiology of recurrent stroke - case report
Source: J Vasc Bras. 2025 Mar 14;24:e20240126. doi: 10.1590/1677-5449.202401262 (PMC11922328; doi:10.1590/1677-5449.202401262)
Supplement: Translation

# Translation: Membrana carotídea: uma etiologia pouco conhecida de acidentes vasculares cerebrais recorrentes - relato de caso

**DOI:** 10.1590/1677-5449.20240126

**Authors:** Nathalia Santana Moreda, Henrique Alves de Almeida, Vinicius Tadeu Ramos da Silva Grillo

**Affiliations:** 1 Centro Universitário São Lucas – UNISL, Porto Velho, RO, Brasil.; 2 Instituto Vascular e Endovascular de Rondônia – IVER, Porto Velho, RO, Brasil.

**Keywords:** doenças das artérias carótidas, AVC isquêmico, endarterectomia de carótida

## Abstract

A membrana carotídea (MC) é uma condição rara e pouco compreendida associada a acidente vascular isquêmico (AVCi) criptogênico e recorrente em pacientes jovens sem fatores de risco ateroscleróticos. Descrevemos o caso de uma paciente do sexo feminino, com 58 anos, hipertensa, dislipidêmica e com quadro de AVCis há 5 anos. A presença de MC foi sugerida pela ultrassonografia Doppler e confirmada pela angiografia por subtração digital. A paciente foi submetida a endarterectomia e, devido ao refluxo satisfatório da carótida interna, optou-se por não utilizar *shunt* vascular temporário. Procedeu-se com a endarterectomia e arteriorrafia com *patch* de pericárdio bovino. Embora a MC possa ser visualizada por meio de exames de imagem, sua detecção pode ser desafiadora, podendo mimetizar outras condições, como dissecção arterial, placa aterosclerótica não calcificada e trombo intraluminal. A facilidade com que a MC pode passar despercebida ou ser mal diagnosticada ressalta a necessidade da compreensão desta doença.

## INTRODUÇÃO

A determinação da etiologia do acidente vascular cerebral isquêmico (AVCi) agudo é crucial para orientar estratégias de tratamento e prevenção secundária. O AVC isquêmico (AVCi) criptogênico representa um desafio significativo para o médico especialista, constituindo aproximadamente 25% de todos os casos de AVCi. Esse subtipo de acidente vascular cerebral demonstra um risco cumulativo elevado de 21,3% para recorrência, indicando a persistência da causa subjacente^1,2^ .

A membrana carotídea (MC), também conhecida como *carotid web*, é uma condição rara e pouco compreendida associada a AVCi criptogênico e recorrente em pacientes jovens desprovidos de fatores de risco ateroscleróticos^1,3,4^ . Essa patologia representa uma forma incomum de displasia fibromuscular focal, caracterizada pela projeção anormal de um septo membranoso na camada interna da carótida, localizado na face posterior do bulbo carotídeo e da artéria carótida interna^3-5^ .

O septo formado pela MC propicia distúrbios hemodinâmicos que causam estagnação do fluxo, favorecendo a formação de trombos e culminando em embolia cerebral. Essas condições indicam que a presença de uma rede carotídea pode estimular a trombogênese, elevando o risco de AVCi^1,3,6^ .

A prevalência da MC na população em geral não é conhecida, mas, entre pacientes com AVCi, varia de 1,2% a 2,7%. A incidência é de 8,9% em pacientes com ataque isquêmico transitório (AIT), 1,1% em pacientes com AVC por oclusão de grandes vasos, e 4,4% em pacientes submetidos a endarterectomia devido a estenose carotídea^2,3,7,8^ .

A MC é mais frequente no sexo feminino, que representa entre 61,7 e 71% dos casos. Afeta predominantemente a população negra, a qual pode corresponder a 75% dos casos, especialmente mulheres jovens com média de idade de 50 anos^3,9-11^ . Dados de uma recente revisão sistemática revelaram que a MC ocorre bilateralmente em 23,0% dos casos, com a maioria das membranas localizadas na parede posterior (87,3%) da artéria carótida (98,7%). Adicionalmente, 31,6% dos pacientes apresentaram trombo aderido à membrana, e 75,9% apresentavam oclusão de grandes vasos^12^ .

## RELATO DE CASO

Apresentamos o caso de uma paciente do sexo feminino, 58 anos de idade, com histórico de hipertensão arterial sistêmica e dislipidemia por uma década, além de ter se submetido a cirurgia bariátrica. Entre os anos de 2017 e 2022, a paciente apresentou episódios recorrentes de AVCi no território da artéria carótida interna direita (ACID).

Durante os episódios isquêmicos, a paciente foi submetida a uma extensa investigação complementar, incluindo tomografia computadorizada e ressonância magnética intracraniana, ultrassonografia Doppler (USD) das artérias carótidas e ecocardiografia, em busca de focos emboligênicos. Contudo, não foram identificadas alterações significativas, e a etiologia dos eventos foi considerada como criptogênica.

Em 2023, novas avaliações complementares incluíram uma nova USD das artérias carótidas, a qual revelou imagem sugestiva de MC na parede posterior do segmento proximal da ACID. Para confirmar o diagnóstico, a paciente foi submetida a angiografia por subtração digital (ASD) com sequência rotacional tridimensional, que confirmou a presença de MC na ACID (Figura 1). A membrana estava localizada na parede posterior, projetando-se para a luz do vaso, resultando em falha de enchimento nesse local e redução da vascularização no território da artéria cerebral média ipsilateral.

**Figura 1:** Angiografia por subtração digital com cateter posicionado na artéria carótida comum direita evidenciando membrana carotídea, que se inicia na carótida comum e se estende para a origem da carótida interna. À direita: angiografia em anteroposterior com referência óssea. À esquerda: angiografia sem referência óssea.

Após a avaliação de risco cirúrgico, a paciente, classificada como baixo risco, foi submetida a endarterectomia da ACID. O procedimento foi conduzido sob anestesia geral com intubação orotraqueal, acompanhada de antibioticoprofilaxia utilizando cefalosporina de primeira geração. Iniciou-se com cervicotomia longitudinal à direita, seguida pela dissecção e reparo da artéria carótida comum (ACC), carótida interna (ACI), e carótida externa (Figura 2). Foi realizada heparinização sistêmica com 5.000 UI de heparina não fracionada endovenosa.

**Figura 2:** Fotografia intraoperatória após dissecção e reparo com *vessel-loop* da artéria carótida comum (à direita da imagem), artéria carótida externa (parte superior esquerda da imagem), artéria tireóidea superior (parte superior da imagem) e artéria carótida interna (parte inferior da imagem).

A arteriotomia foi efetuada da ACC até a ACI, e, devido ao refluxo satisfatório da ACI, optou-se por não utilizar shunt vascular temporário. Durante a arteriotomia, foi possível visualizar o flap membranoso no bulbo carotídeo (Figura 3). Procedeu-se com endarterectomia e retirada da MC e, para a arteriorrafia, foi utilizado *patch* de pericárdio bovino (Figura 4).

**Figura 3:** Fotografia intraoperatória após clampeamento da artéria carótida comum interna e externa e arteriorrafia. Observa-se flap membranoso no bulbo carotídeo.

**Figura 4:** Fotografias do intraoperatório. À esquerda: ressecção da membrana carotídea. À direita: arteriorrafia com implante de *patch* de pericárdio bovino.

A paciente foi transferida para a unidade de terapia intensiva (UTI) para os cuidados pós-operatórios e progrediu sem a ocorrência de alterações neurológicas ou complicações no sítio cirúrgico. Após 1 dia de internação na UTI, a paciente foi transferida para um leito de enfermaria e permaneceu mais 1 dia, culminando na alta hospitalar em excelente estado geral. Nas reavaliações subsequentes, a paciente permaneceu assintomática do ponto de vista neurológico, e a USD de controle evidenciou a perviedade das carótidas.

Em angiorresonância magnética arterial dos vasos cervicais realizada após um ano da cirurgia, observou-se área de encefalomalácia frontotemporal à direita, compatível com sequela dos eventos isquêmicos prévios. Além disso, a ACID permanece pérvia, com discreta redução do calibre sem determinar estenose significativa, condizente com o pós-operatório (Figura 5).

**Figura 5:** Angiorresonância arterial dos vasos cervicais após 1 ano da cirurgia. Nota-se que a artéria carótida interna direita permanece pérvia, com discreta redução do calibre sem determinar estenose significativa.

## DISCUSSÃO

Inicialmente a etiologia do AVC na paciente foi categorizada como criptogênica. A incidência de AVC criptogênico associado a MC foi de 3,8/100.000 pessoas-ano (3,2 em homens e 4,3 em mulheres), sendo que a incidência de MC foi 24 vezes maior naqueles com AVCi de circulação anterior uni-hemisférica do que em pacientes controle^1^ . Outro estudo observou associação estatisticamente significativa entre MC e AVCi, com uma prevalência 21,2% na população com AVC criptogênico, em oposição a 1,6% no grupo controle, produzindo uma razão de probabilidade de 10,6^11^ .

Além disso, no contexto deste relato, a ocorrência de AVCis recorrentes chamou a atenção, devido ao fato de a paciente ser jovem e apresentar poucos fatores de risco para ateromatose. A MC aparentou ser um importante fator de suspeição, uma vez que está associada a um risco cinco vezes maior de recorrência de AVC em comparação aos casos sem essa condição^5^ . Além disso, os fatores de risco habituais na maior parte dos casos de AVC foram menos prevalentes nos casos de AVC por MC, destacando-se a presença de hipertensão em 28,6%, hiperlipidemia em 14,6%, diabetes mellitus em 7,0%, e tabagismo em 19,8%^9^ .

A confusão diagnóstica pode surgir, mesmo durante avaliações realizadas por profissionais experientes, devido à rara ocorrência da MC, que frequentemente é confundida com dissecção arterial espontânea e placas ateroscleróticas^3^ . Essa complexidade no diagnóstico destaca a necessidade de uma abordagem cuidadosa e a consideração de causas menos comuns em casos de apresentações atípicas, reforçando a importância do conhecimento detalhado das características distintivas da MC para uma avaliação diagnóstica mais precisa.

A USD das carótidas possibilita a visualização luminal e a identificação da MC, nos casos com ou sem estenose significativa. Contudo, a angiotomografia computadorizada (ATC) e a ASD surgem como ferramentas mais vantajosas na detecção da MC, permitindo a observação de estagnação de contraste no septo membranoso, mesmo na fase venosa tardia^1,3,12^ .

Embora a MC possa ser visualizada por meio de ATC e ASD, sua detecção pode ser desafiadora, uma vez que geralmente não causa estenose hemodinamicamente significativa. Adicionalmente, a MC pode mimetizar outras condições, como dissecção arterial, placa aterosclerótica não calcificada e trombo intraluminal^1,12^ . No caso em questão, a paciente foi submetida a exames de imagem intracraniana, os quais não abrangeram a avaliação dos vasos cervicais, possivelmente contribuindo para o atraso no diagnóstico. Sajedi et al.^11^ propõem a vantagem da ATC, devido à obtenção rápida e alta resolução de imagens de forma não invasiva, além de seu benefício adicional na caracterização de achados sobrepostos e de auxiliar na exclusão de aterosclerose ou lesões vasculares; assim, os autores recomendam que pacientes com AVC criptogênico recebam imagens de ATC do pescoço como parte da avaliação padrão.

A melhor abordagem terapêutica para a MC ainda não foi definida com base em evidências robustas, embora seja reconhecido que o tratamento medicamentoso pode não ser suficiente para reduzir os eventos isquêmicos recorrentes^3-5^ .

A paciente do caso clínico foi submetida com sucesso a endarterectomia de carótida, destacando-se como uma opção viável para o tratamento da MC. Uma revisão bibliográfica recente comparando tratamento médico e intervencionista em 289 pacientes com MC revelou que 52,2% foram submetidos a intervenção (angioplastia ou endarterectomia), enquanto 47,8% foram tratados clinicamente (antiagregação ou anticoagulação). No grupo de intervenção, não houve mortalidade periprocedimento, e complicações maiores foram registradas em 0,5%, com ausência de eventos isquêmicos recorrentes durante o acompanhamento de 3 a 60 meses. Em contraste, no grupo de tratamento médico, ao longo de 2 a 55 meses, a taxa de recorrência de isquemia cerebral foi significativamente maior, atingindo 26,8%^9^ .

## CONCLUSÃO

A MC, uma anomalia rara de diagnóstico desafiador, destaca-se como uma condição que favorece o risco de AVCi, principalmente aqueles recorrentes e em pacientes jovens sem fatores de risco ateroscleróticos.

A celeridade na elucidação diagnóstica é fundamental para um prognóstico otimizado, considerando a potencial gravidade associada à patologia. A facilidade com que pode passar despercebida ou diagnosticada tardiamente ressalta a necessidade imperativa de os profissionais de saúde reforçarem sua aprendizagem e compreensão dessa doença. A abordagem individualizada e o domínio dos exames complementares desempenham papel crucial no diagnóstico precoce e tratamento eficaz dessa condição complexa.
